# PlaASDB: a comprehensive database of plant alternative splicing events in response to stress

**DOI:** 10.1186/s12870-023-04234-7

**Published:** 2023-04-27

**Authors:** Xiaokun Guo, Tianpeng Wang, Linyang Jiang, Huan Qi, Ziding Zhang

**Affiliations:** 1 State Key Laboratory of Farm Animal Biotech Breeding, College of Biological Sciences, China Agricultural University, Beijing, 100193 China; 2 State Key Laboratory of Molecular Developmental Biology, Institute of Genetics and Developmental Biology, Chinese Academy of Sciences, Beijing, 100101 China

**Keywords:** Plant, Alternative splicing, RNA-Seq data, Stress response, Arabidopsis, Rice

## Abstract

**Background:**

Alternative splicing (AS) is a co-transcriptional regulatory mechanism of plants in response to environmental stress. However, the role of AS in biotic and abiotic stress responses remains largely unknown. To speed up our understanding of plant AS patterns under different stress responses, development of informative and comprehensive plant AS databases is highly demanded.

**Description:**

In this study, we first collected 3,255 RNA-seq data under biotic and abiotic stresses from two important model plants (Arabidopsis and rice). Then, we conducted AS event detection and gene expression analysis, and established a user-friendly plant AS database termed PlaASDB. By using representative samples from this highly integrated database resource, we compared AS patterns between Arabidopsis and rice under abiotic and biotic stresses, and further investigated the corresponding difference between AS and gene expression. Specifically, we found that differentially spliced genes (DSGs) and differentially expressed genes (DEG) share very limited overlapping under all kinds of stresses, suggesting that gene expression regulation and AS seemed to play independent roles in response to stresses. Compared with gene expression, Arabidopsis and rice were more inclined to have conserved AS patterns under stress conditions.

**Conclusion:**

PlaASDB is a comprehensive plant-specific AS database that mainly integrates the AS and gene expression data of Arabidopsis and rice in stress response. Through large-scale comparative analyses, the global landscape of AS events in Arabidopsis and rice was observed. We believe that PlaASDB could help researchers understand the regulatory mechanisms of AS in plants under stresses more conveniently. PlaASDB is freely accessible at http://zzdlab.com/PlaASDB/ASDB/index.html.

**Supplementary Information:**

The online version contains supplementary material available at 10.1186/s12870-023-04234-7

## Background

Plants inevitably encounter different environmental stresses during their survival, including abiotic stress (e.g., heat stress and drought stress) and biotic stress (e.g., infections of viruses, bacteria, fungi, and parasites). Due to their sessile lifestyle, plants need to rapidly and accurately adjust their transcriptional regulations, including the altered expression levels of transcripts and the changes in alternative splicing (AS) patterns, to generate unique and highly coordinated molecular responses under different environmental factors [1–3].

AS is a co-transcriptional regulatory mechanism which can regulate the recognition of splice sites, resulting in multiple transcripts for each gene [4, 5]. These transcripts further contribute greatly to the diversity of transcriptome and proteome. On the one hand, AS functions primarily by producing two or more protein isoforms, which may perform completely different functions in certain situations [6]. On the other hand, AS can also lead to the functional loss of genes, as it can result in premature termination codons (PTCs) and yield the production of truncated protein isoforms [7]. PTCs caused by AS may further trigger nonsense-mediated decay (NMD), leading to cytoplasmic RNA degradation [8, 9]. AS events contain four major types, including intron retention (IR), exon skipping (ES), alternative 5' splice sites (A5SS) and alternative 3' splice sites (A3SS) [10]. IR is the most commonly occurred AS events in plants, which is different from animals.

With the development of sequencing technology, the detection of AS events from RNA-Seq data has become more mature, and a large amount of data associated with AS have been accumulated in public databases. There are several AS-specific databases for plants, such as CuAS [11], PASTDB [7], and TeaAS [12], which are playing increasingly important roles in accelerating our understanding of AS patterns in these plant species. However, most of these databases focus on only one individual species. In general, global and systematic comparison of AS events in plants is still lacking, largely due to the absence of highly integrated data resource. Meanwhile, there are more and more AS studies on plants, but most still focus on single genes or the whole transcriptome in response to specific environmental conditions [13]. Therefore, it is necessary to jointly investigate the AS events of different species under diverse stresses through the construction of more comprehensive AS databases and the large-scale analysis of AS events. In the past decades, RNA-Seq data in *Arabidopsis thaliana* (Arabidopsis) and *Oryza sativa* (rice) have been accumulated rapidly as they are two of the most important model plants. Although specific databases designed to manage the RNA-Seq data of these two plants have been available, the detection of AS events from the RNA-Seq data are often overlooked. For instance, there is currently an Arabidopsis RNA-Seq database called ARS, which contains about 20,000 samples in Arabidopsis, but it does not target the AS events [14].

In this study, a large number of RNA-Seq data of Arabidopsis and rice were collected to investigate their AS patterns under biotic and abiotic stresses in detail. We pre-calculated and integrated AS events and gene expression patterns under different stress conditions as well as the related genomic characteristics into a comprehensive plant-specific AS database termed PlaASDB. We also compared AS patterns between Arabidopsis and rice under stresses with the data from PlaASDB. Through these large-scale comparisons, the global landscape of AS events in Arabidopsis and rice was observed, the differential splicing and differential expression of genes induced by different stresses were compared, and the possible functions and mechanisms caused by the AS changes were also discussed.

## Results and discussion

### Database construction

**Fig. 1:**
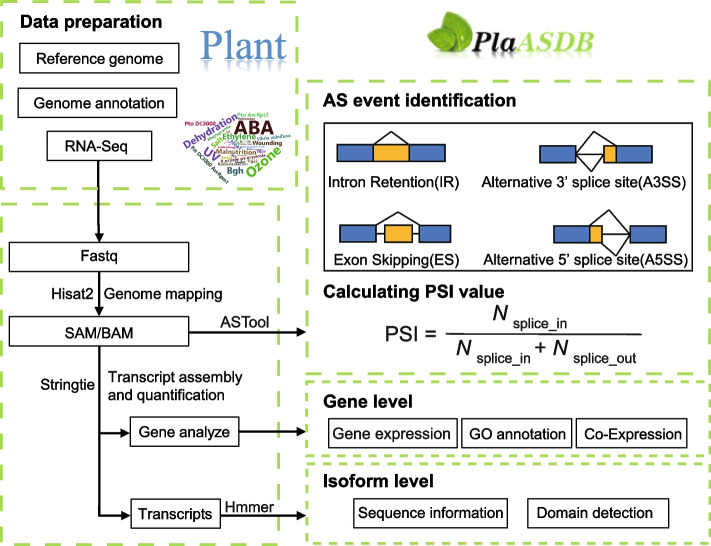
The work flow of PlaASDB. The stress-related RNA-Seq data of Arabidopsis and rice were collected and processed with a standardized pipeline. Then, the expression values of genes and transcripts were calculated, and AS events were identified uniformly by using ASTool. In addition, co-expression networks of genes were constructed, and the related annotation information was also collected

We collected 2,703 RNA-Seq data sets of Arabidopsis, including 2,280 abiotic and 423 biotic stress samples, and 552 RNA-Seq data sets of rice, including 410 abiotic and 142 biotic stress samples (see Table S1 for more details). We processed these RNA-Seq data uniformly and constructed an AS-specific database termed PlaASDB. The workflow of PlaASDB construction is shown in Fig. 1. We used ASTool [15], an exon-based AS detection tool developed by our team, to identify four major AS events (IR, ES, A5SS, and A3SS) in different samples.

**Fig. 2:**
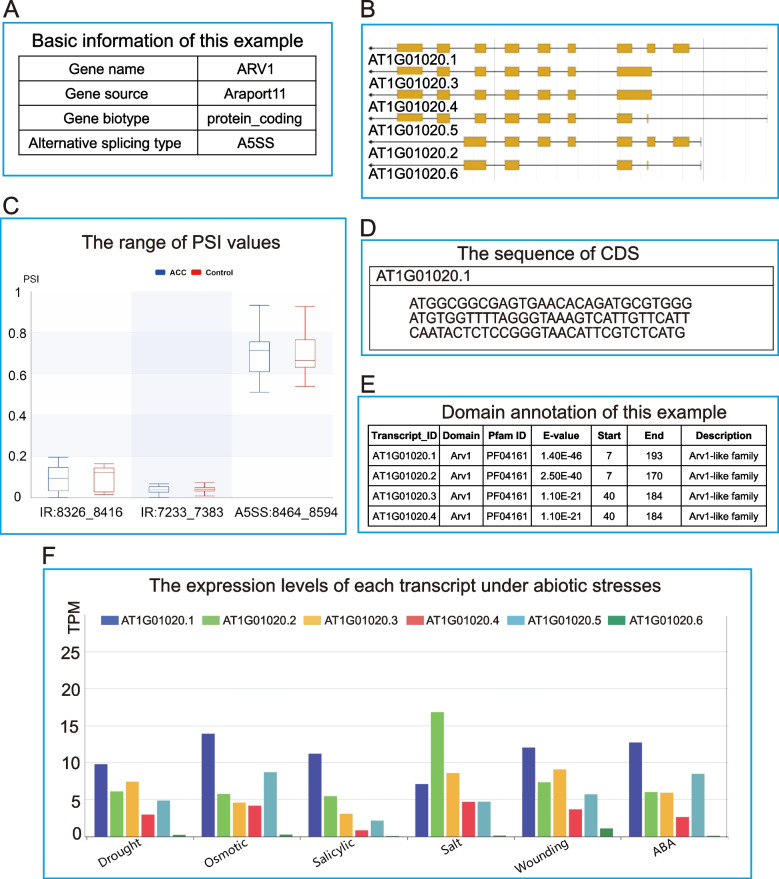
A brief introduction of PlaASDB through an example (gene ID: AT1G01020). **A** Basic information about the example. **B** Database built-in gene browser. **C** PSI range of AS events under 1-aminocyclopropane-1-carboxylic acid (ACC) stress. **D** The basic information of the transcript sequence. **E** Information of domains detected from transcripts. **F** The average transcripts per million reads (TPM) values of the transcripts in each stress type. For each stress type, we removed the control samples, calculated the TPM values of the transcripts from multiple experiments using StringTie (v2.1.4), and then obtained the average TPM value as the expression level of each transcript

The PlaASDB database mainly deposits the AS information of Arabidopsis and rice genes in response to multiple abiotic and biotic stresses, and other basic transcriptome information is also integrated. As shown in Fig. 2, we show the usage of PlaASDB through the query results of an Arabidopsis gene named *ARV1* (gene ID: AT1G01020). As a protein-coding gene, it is often active in the golgi apparatus and cortical endoplasmic reticulum, and plays an important role in the metabolic processes of sphingolipids and sterols. This gene contains six different transcripts. PlaASDB provides a retrieval system enabling users to search by gene ID (AT1G01020) or name (*ARV1*). It should be noticed that there are two kinds of rice gene IDs, RGAP and RAP-DB. Users can choose any type of them to complete the query without the need for ID conversion. After searching, PlaASDB first provided basic information about the target gene, including the types of AS events that the gene may be involved in under different stress conditions (Fig. 2A). At the same time, users can visualize the target gene in the built-in gene browser, in which users can also query according to their demands. For example, users can further click on each transcript to view the relevant information (Fig. 2B). The PlaASDB database then provides detailed information of all AS events, including the type, length, location, and the average percent-splice-in (PSI) value. Users can select the specific stress type they are interested in and visualize the corresponding PSI range of AS events in the target gene (Fig. 2C). Users can obtain the information on transcripts, CDS, and isoform sequences directly from the resulting page (Fig. 2D). For different isoforms, the Pfam domain annotations are also listed (Fig. 2E). The expression levels of genes and transcripts under different stress types were also available (Fig. 2F). Finally, we calculated the Pearson correlation coefficients (PCCs) between the gene expression profiles of the target gene and other genes, and inferred the co-expressed gene partners of the target gene according to the ranking of PCCs. Either in abiotic or biotic stress, the top 50 co-expressed genes of the target gene are displayed. To help users understand the potential functionality of the target gene, PlaASDB also provides short descriptions of the co-expressed genes and the corresponding links to other databases. Further using AT1G01020 as an example, its two co-expression networks under abiotic and biotic stresses are visualized in Fig. S1. Altogether, PlaASDB can provide some new clues to interrogate the functional roles of the query gene in abiotic and biotic stresses by taking the AS events and the co-expression partners simultaneously into account.

#### Identification and analysis of gene isoforms

**Fig. 3:**
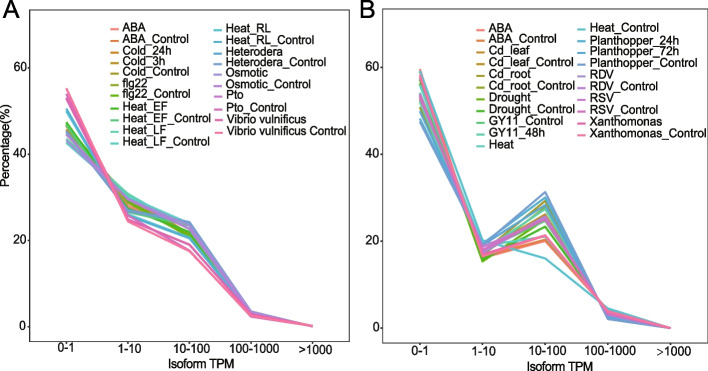
Transcript abundance of Arabidopsis (**A**) and rice (**B**) under different stress conditions

Gene isoforms are multiple copies of mRNAs from one gene but differ in transcription products, potentially altering gene function. Representative RNA-Seq data of Arabidopsis and rice under different biotic and abiotic stress types were selected from PlaASDB for further analysis, including 57 Arabidopsis and 63 rice samples, respectively (see Table S2 for more details). In addition, these samples were collected from multiple tissues of Arabidopsis and rice, such as roots, leaves, and seeds (Table S2). A total of 54,013 isoforms were identified in Arabidopsis and 66,338 isoforms in rice, respectively. Results suggested that low-abundant transcripts (i.e., transcripts per million reads (TPM) < 1) occupied the largest proportion in Arabidopsis and rice (Fig. 3). Most of the low-abundant transcripts cannot be successfully translated into proteins [16, 17]. This may explain why the number of proteins is much smaller than the number of transcripts. At the same time, for Arabidopsis and rice, the major isoforms (e.g., the top 1 and 2 isoforms) contribute the majority of the gene expression abundance, while the remaining isoforms cover a small proportion of the gene expression abundance. The results indicated that the top 1 and top 2 isoforms often play a dominant role in gene function.

#### Characterization of global AS events in Arabidopsis and rice

**Fig. 4:**
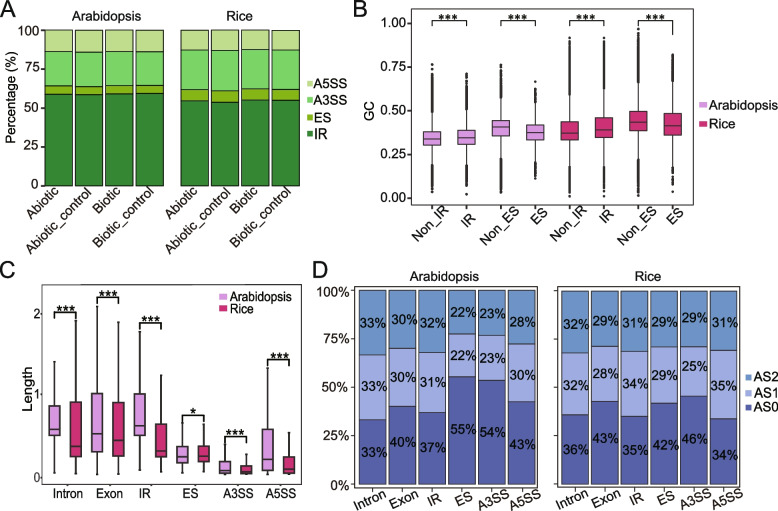
Alternative splicing (AS) events in Arabidopsis and rice under stress. **A** The proportion of four types of AS events in Arabidopsis and rice. **B** The GC content of the retained introns and the skipped exons in Arabidopsis and rice are compared. **C** Length distribution of genome-wide introns, exons, and four major kinds of AS events in Arabidopsis and rice (*** means *P*-value <0.01, * means *P*-value <0.05). All lengths were divided by the average length of the corresponding introns/exons in the whole genome for standardization. **D** Distribution in ASX_i_ types and their frequency in Arabidopsis (left) and rice (right). ASX_i_ stands for the remainder of the length of an intron or exon divided by 3. X_i_ ∈ {0,1,2}

We further investigated the AS patterns in Arabidopsis and rice under stress with the selected 120 RNA-Seq samples. A total of 9,052 and 16,680 AS events were identified in Arabidopsis and rice, respectively. As shown in Fig. 4A, IR was the dominant AS event in Arabidopsis and rice, followed by A3SS, A5SS, and ES. The proportion of A3SS events is much higher than that of A5SS events, which is consistent with previous reports [4, 18, 19]. Interestingly, we found a higher percentage of IR in Arabidopsis than in rice, while the proportion of A3SS in rice was higher than in Arabidopsis (Fig. 4A, Fig. S2). Regarding the proportions of AS events detected under stress and control conditions, no significant difference was observed.

We also compared the GC content of locus detected with AS events, including retained introns (PSI ≥ 0.1) and skipped exons (PSI ≤ 0.9), with locus without AS events (Fig. 4B). GC content calculation of A5SS and A3SS was not conducted because the sequence lengths are too short. The GC content of retained introns was significantly higher than other introns in Arabidopsis and rice (two-tailed Wilcox-test, *P*-value < 0.001). Meanwhile, the GC content of skipped exons was significantly lower than that of constituent exons (two-tailed Wilcox-test, *P*-value < 0.001). Therefore, when the GC content is low in exons, exon skipping events are more likely to occur. On the contrary, when the GC content is high in introns, it is more likely to retain these introns in mature transcripts. These indicated that GC content could be a vital feature in differentiating AS events [20, 21].

We further analyzed the length distribution of AS events. In Arabidopsis and rice, the length distribution patterns of the four AS events are approximately the same. The average lengths of IR, ES, A5SS, and A3SS events are about 190 bp, 110 bp, 150 bp, and 70 bp in Arabidopsis, and 300 bp, 150 bp, 120 bp, and 70 bp in rice, respectively. We then compared the length of the four AS events and genome-wide introns and exons in Arabidopsis and rice (Fig. 4C), and found significant differences (two-tailed Wilcox test, *P*-value < 0.001) in four AS events, which may also be related to the evolution of rice genome. We further investigated the effect of the length of AS events on the reading frame and found that Arabidopsis and rice are inclined to keep the reading frame when faced with evolutionary pressure (Fig. 4D).

#### Identification and functional analysis of DSGs and DEGs under biotic and abiotic stresses

**Fig. 5:**
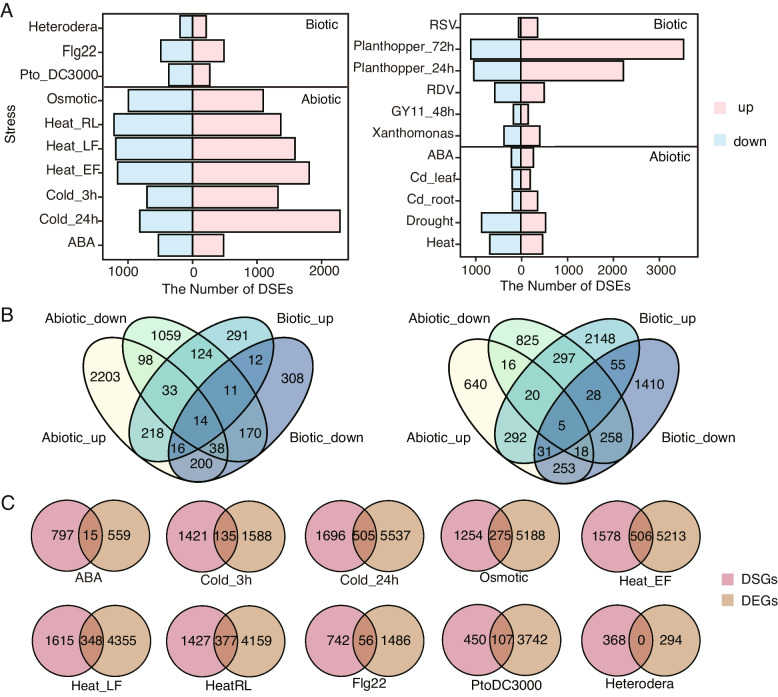
**A** Differential splicing events (DSE) between Arabidopsis (left) and rice (right) under different stresses. **B** Venn diagram analysis of up- and down-regulated differential splicing events under biotic and abiotic stresses in Arabidopsis (left) and rice (right). **C** Venn diagram of differentially expressed genes (DEGs) and differentially spliced genes (DSGs) in Arabidopsis under different stresses

We first calculated the PSI values of AS events using ASTool [15]. Then, we identified differential AS events under biotic and abiotic stresses with the following two criteria: (1) |ΔPSI|≥ 0.1, and (2) *P*-value based on two-tailed Wilcox-test < 0.1. We defined an AS event with changed PSI under stress as an up-regulated or down-regulated one. We first compared the number of differential AS events of Arabidopsis and rice under different stresses (Fig. 5A). For Arabidopsis, we detected more differential AS events under abiotic stresses than biotic stresses. The overall fluctuation of differential AS events under abiotic stress was large. Except for ABA and osmotic stress, the up-regulated differential AS events were far more than the down-regulated differential AS events. However, the number of differential AS events for rice was more under biotic stresses than that under abiotic stresses. Results indicated more down-regulated differential AS events under abiotic stresses, such as drought and heat (Fig. 5A). In particular, the number of up-regulated differential AS events was more than twice the down-regulated ones in rice infected by Planthopper. We further investigated each type of differential AS events under both abiotic and biotic stresses (Fig. 5B). It was found that some differential AS events occurred simultaneously under abiotic and biotic stresses in both Arabidopsis and rice. We assumed that these events conservatively regulate plants through AS mechanisms to better adjust to the environment.

We then referred to genes containing differentially spliced AS events as DSGs. We also identified differentially expressed genes (DEGs) under stresses with the R package DESeq2 [22] to compare the difference between DSGs. As a result, we identified 1,435 DSGs under biotic stress and 4,184 DSGs under abiotic stress in Arabidopsis. Similarly, we identified 4,815 DSGs under biotic stress and 2,683 DSGs under abiotic stress in rice. For DEGs, 4,704 DEGs and 12,061 DEGs were identified under biotic and abiotic stresses in Arabidopsis, 11,403 DEGs under biotic stress and 17,439 DEGs under abiotic stress were identified in rice, separately (Table S3). In contrast, under each stress, there were only limited DSGs also detected as DEGs in both Arabidopsis (Fig. 5C) and rice (Fig. S3), indicating that gene expression regulation and AS seemed to play independent roles in response to stresses [7].

In order to have a better understanding of the functions of DSGs and DEGs, we conducted gene ontology (GO) enrichment analysis (Tables S4 and S5) [23]. In Arabidopsis and rice, DSGs are enriched in many of the same GO terms. For example, in the category of biological process, we noticed that several broad GO terms were commonly enriched among DSGs in Arabidopsis and rice under abiotic and biotic stresses, such as regulation of cellular process and regulation of biological process. Regarding the category of molecular function, GO terms were mainly concentrated on binding-related terms, such as nucleotide binding and ATP binding. These results are consistent with previous studies of plant AS events in response to some specific stresses [24].

For DEGs in response to biotic stress, GO terms responding to stresses were significantly enriched in both Arabidopsis and rice, including response to stress, response to abiotic stimulus, cellular response to stimulus, and response to oxidative stress. However, we found the number of commonly enriched terms in DEGs was far less than in DSGs when under abiotic stresses (Tables S4 and S5), indicating DSGs might potentially play more conservative roles in plants. In molecular function, the terms related to binding-related function were also enriched as shown in DSGs (Tables S4 and S5).

Differences in biological processes between DSGs and DEGs indicated that AS might play an important role in the stress response by affecting gene groups other than conventional DEGs [19, 25]. For example, the *REGULATOR OF CBF EXPRESSION*1 (*RCF*1, AT1G20920) encodes an RNA helicase required for cold tolerance [26]. According to the annotation of this gene in PlaASDB, *RCF*1 contains mRNA splicing and RNA helicase activity-related GO terms. Moreover, it contains seven transcripts and undergoes IR, ES, A5SS and A3SS events under cold stress. Interestingly, previous experimental evidence indicates that AS of the introns in the 3′ untranslated region (UTR) may lead to the retention of a specific isoform in the nucleus or trigger NMD to regulate *RCF*1 expression at different temperatures [27]. At the same time, both DSGs and DEGs play a central functional role in transcriptional regulatory networks and are potentially pleiotropic [28]. We also found many specific enriched GO terms in each plant under abiotic or biotic stresses, enabling further investigation into the functions of these DSGs or DEGs.

## Conclusions

As a universal transcriptional regulatory mechanism, AS plays an important role when plants cope with environmental stress. The application of RNA-Seq technology continuously promotes the development of plant transcriptomics in recent years, and researchers could obtain more AS information through the analysis of transcripts. PlaASDB is a freely available public resource that provides extensive details of AS events by analyzing RNA-Seq data of two important model plants (Arabidopsis and rice) under biotic and abiotic stresses. The established PlaASDB also allowed us to obtain the global landscape of AS events between Arabidopsis and rice under biotic and abiotic stresses. Moreover, the DSGs and DEGs of these two plants in response to different stress types were also systematically investigated. Taken together, we hope that PlaASDB will become an important resource to investigate AS patterns in plants under different stresses and thus provides new hints to accelerate the functional genomics studies of plants.

## Methods

### Data collection and process

We collected stress-related RNA-Seq metadata of Arabidopsis thaliana (Arabidopsis) and Oryza sativa(rice) from Gene Expression Omnibus (GEO) database [29] and downloaded the corresponding raw data from NCBI Sequence Read Archive (SRA) database [30]. In total, we obtained an RNA-Seq dataset consisting of 3,255 samples from Arabidopsis and rice (Table 1 and Table S1). After quality control of raw data through Trimmomatic (v0.39) [31], we used Hisat2 [32] to align reads to the reference genome with default parameters. Then, the SAM file from Hisat2 was converted to a BAM file using Samtools (v1.11) [33]. Moreover, the transcripts were assembled according to reference genomes, and the corresponding expression levels (i.e., TPM values) were calculated by using StringTie (v2.1.4) [34]. Finally, ASTool [15] was used to identify AS events for each sample through the calculation of the corresponding PSI values.

**Table 1:** Brief information of collected samples in PlaASDB

Species	Stress types	No. of projects	No. of samples
Arabidopsis	Abiotic	107	2280
	Biotic	18	423
Rice	Abiotic	32	410
	Biotic	16	142

We have classified these samples according to stress conditions, and detailed information on these RNA-Seq data is summarized in Table S1. We further selected representative samples of Arabidopsis and rice under several abiotic and biotic stress conditions from the compiled database for subsequent analysis of AS events (Table S2). To avoid the influence of ecotypes and mutants, representative samples should be Col-0 wild-type. Additionally, three biological repeats were required in most representative samples.

#### Gene information collection and functional annotation

We downloaded gene information, transcripts, and protein sequences of Arabidopsis from TAIR (https://www.arabidopsis.org/) [35]. We also collected the corresponding information on rice from RAP-DB (https://rapdb.dna.affrc.go.jp/) [36]. GO annotations of Arabidopsis and rice were downloaded from the GO database (http://geneontology.org/) [23]. We used HMMER (v3.3.2) [37] to search for domains in each transcript in the Pfam database with an E-value threshold of 1E-5.

#### Co-expression network construction

Conditional-specific gene co-expression networks were constructed for each project. Only the projects containing at least 12 samples were considered. For each project, we calculated the value of PCC between any two genes [38]. To construct the co-expression networks of a gene, the top 50 co-expressed gene partners for this gene were selected. Finally, all the top 50 co-expressed gene partners of this gene from different projects were integrated to obtain the co-expression networks of this gene under biotic stress and abiotic stress. In the co-expression network under biotic or abiotic stress, note that only the top 50 co-expressed genes after ranking were considered.

#### Identification of DSGs and DEGs

We used ASTool to calculate the PSI value for each AS event. The change of PSI value (∆PSI) was used to measure the degree of difference in retained intron and skipped exon. We calculated the ∆PSI values by evaluating the difference between the average PSI values of the treated and control samples. In addition, *P*-value was used to measure the significance of the difference in ∆PSI values with a nonparametric Wilcoxon rank sum test. Differential AS events were defined with the following thresholds: |ΔPSI|≥ 0.1 and *P*-value ≤ 0.1. Genes containing differential AS events were defined as DSGs. We also identified DEGs under different kinds of stresses with the R package “DESeq2” [22]. Genes with |log_2_FC|≥ 1 and adjusted *P*-value < 0.05 were considered as DEGs.

#### Gene ontology enrichment analysis

Gene functions of DSGs and DEGs were annotated according to the GO database [23]. GO enrichment analysis was performed by agriGO (v2.0) [39]. Enriched GO terms with adjusted *P*-value < 0.05 were selected for further comparison.

#### Database construction

The PlaASDB website was built based on CentOS 7.4, Apache 2.4.6, MySQL 15.1, and PHP 5.4.16. JavaScript was used for document manipulating, data visualization, and the built-in gene browser. The tables and charts in PlaASDB were mainly produced based on several web-based JavaScript libraries, such as DataTables.js and echarts.js.

## Supplementary Information

**Additional file 1: Table S1.** The detailed information of all RNA-seq samples in PlaASDB. **Table S2.** The 120 RNA-seq samples used for analysis. **Table S3.** The number of DEGs and DSGs in Arabidopsis and rice under different stresses. **Table S4.** The enriced GO terms of DSGs and DEGs in Arabidopsis. **Table S5.** The enriched GO terms of DSGs and DEGs in rice. **Figure S1.** The established abiotic stress (A) and biotic stress (B) co-expression networks of AT1G01020. For the convenience ofvisualization, only the top 20 co-expressed genes are shown. **Figure S2.** The percentage of IR, ES, A3SS and A5SS events in samples from Arabidopsis and rice under abiotic stress, biotic stress and control groups. **Figure S3.** Venn diagram showing the overlapping DSGs and DEGs in rice under different stresses.

## Data Availability

PlaASDB is freely available at http://zzdlab.com/PlaASDB/ASDB/index.html. The.dataset can be downloaded from http://zzdlab.com/PlaASDB/ASDB/download.html. The detailed user manual is available at http://zzdlab.com/PlaASDB/ASDB/help.php.

## Abbreviations

AS: Alternative splicing
DSG: Differential splicing gene
DEG: Differential expression gene
IR: Intron retention
ES: Exon skipping
A5SS: Alternative 5' splice sites
A3SS: Alternative 3' splice sites
NMD: Nonsense-mediated mRNA decay
PSI: Percent-splice-in
PCC: Pearson correlation coefficient
TPM: Transcripts per million reads
GEO: Gene Expression Omnibus
SRA: Sequence Read Archive
