# Supplementary material for: PlaASDB: a comprehensive database of plant alternative splicing events in response to stress
Source: BMC Plant Biol. 2023 Apr 27;23:225. doi: 10.1186/s12870-023-04234-7 (PMC10134664; doi:10.1186/s12870-023-04234-7)
Supplement: Supplementary file 1 — Additional file 1: Table S1. The detailed information of all RNA-seq samples in PlaASDB. Table S2. The 120 RNA-seq samples used for analysis. Table S3. The number of DEGs and DSGs in Arabidopsis and rice under different stresses. Table S4. The enriced GO terms of DSGs and DEGs in Arabidopsis. Table S5. The enriched GO terms of DSGs and DEGs in rice. Figure S1. The established abiotic stress (A) and biotic stress (B) co-expression networks of AT1G01020. For the convenience ofvisualization, only the top 20 co-expressed genes are shown. Figure S2. The percentage of IR, ES, A3SS and A5SS events in samples from Arabidopsis and rice under abiotic stress, biotic stress and control groups. Figure S3. Venn diagram showing the overlapping DSGs and DEGs in rice under different stresses. [file 12870_2023_4234_MOESM1_ESM.zip › Supplemental_file_1.docx]

Supplementary material

**PlaASDB:** **A comprehensive database of plant alternative splicing events in response to stress**

Xiaokun Guo, Tianpeng Wang*, Linyang Jiang, Huan Qi, Ziding Zhang*

^*^Corresponding authors: ZZ (zidingzhang@cau.edu.cn), and TW (tpengwang@163.com)

The supplemental material contains the following tables and figures.

Table S1 The detailed information of all RNA-seq samples in PlaASDB.

Table S2 The 120 RNA-seq samples used for analysis.

Table S3 The number of DEGs and DSGs in Arabidopsis and rice under different stresses.

Table S4 The enriced GO terms of DSGs and DEGs in Arabidopsis.

Table S5 The enriched GO terms of DSGs and DEGs in rice.

Figure S1 The established abiotic stress (A) and biotic stress (B) co-expression networks of AT1G01020. For the convenience of visualization, only the top 20 co-expressed genes are shown.

Figure S2 The percentage of IR, ES, A3SS and A5SS events in samples from Arabidopsis and rice under abiotic stress, biotic stress and control groups.

Figure S3 Venn diagram showing the overlapping DSGs and DEGs in rice under different stresses.

*Note that Tables S1, S2, S4 and S5 are available in a separate Excel file*

Table S3 The number of DEGs and DSGs in Arabidopsis and rice under different stresses.

| Species | Stress | Type | Number of DSGs | Number of DEGs |
| --- | --- | --- | --- | --- |
| Arabidopsis | ABA | Abiotic | 812 | 574 |
| Arabidopsis | Cold3h | Abiotic | 1556 | 1723 |
| Arabidopsis | Cold24h | Abiotic | 2201 | 6042 |
| Arabidopsis | HeatEF | Abiotic | 2084 | 5719 |
| Arabidopsis | HeatLF | Abiotic | 1963 | 4703 |
| Arabidopsis | HeatRL | Abiotic | 1804 | 4536 |
| Arabidopsis | Osmotic | Abiotic | 1529 | 5463 |
| Arabidopsis | Pto DC3000 | Biotic | 557 | 3849 |
| Arabidopsis | Flg22 | Biotic | 798 | 1542 |
| Arabidopsis | Heterodera | Biotic | 368 | 294 |
| rice | Heat | Abiotic | 1150 | 15067 |
| rice | Drought | Abiotic | 1394 | 8357 |
| rice | Cd_root | Abiotic | 551 | 1496 |
| rice | Cd_leaf | Abiotic | 391 | 553 |
| rice | ABA | Abiotic | 481 | 1241 |
| rice | Xanthomonas | Biotic | 781 | 2470 |
| rice | GY11_48h | Biotic | 161 | 3478 |
| rice | RDV | Biotic | 1074 | 3538 |
| rice | Planthopper24h | Biotic | 3260 | 2628 |
| rice | RSV | Biotic | 1260 | 3538 |


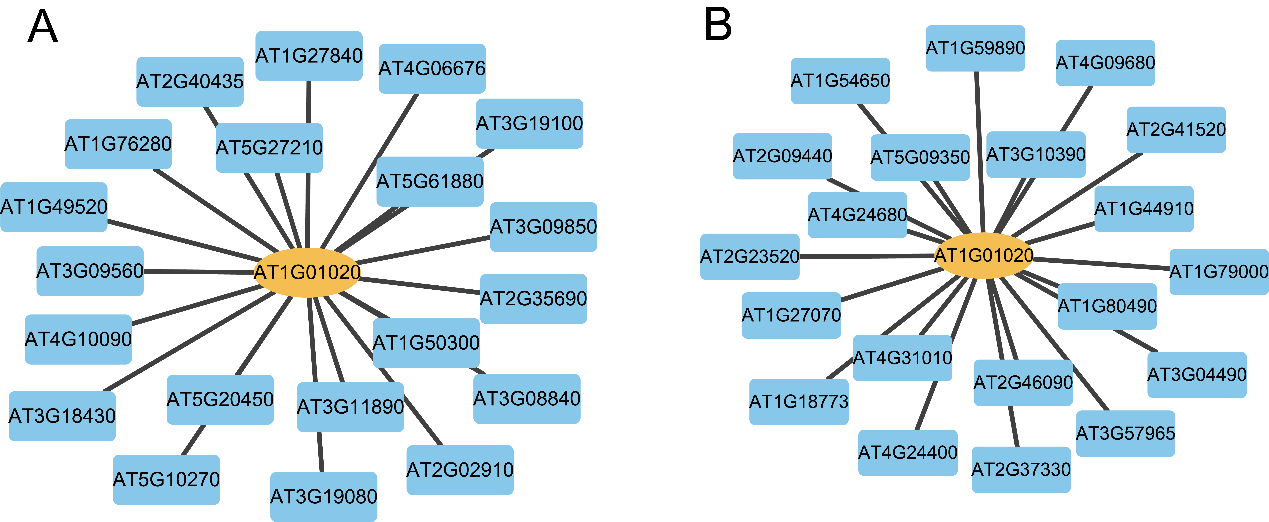


Fig S1. The established abiotic stress (A) and biotic stress (B) co-expression networks of AT1G01020. For the convenience of visualization, only the top 20 co-expressed genes are shown.


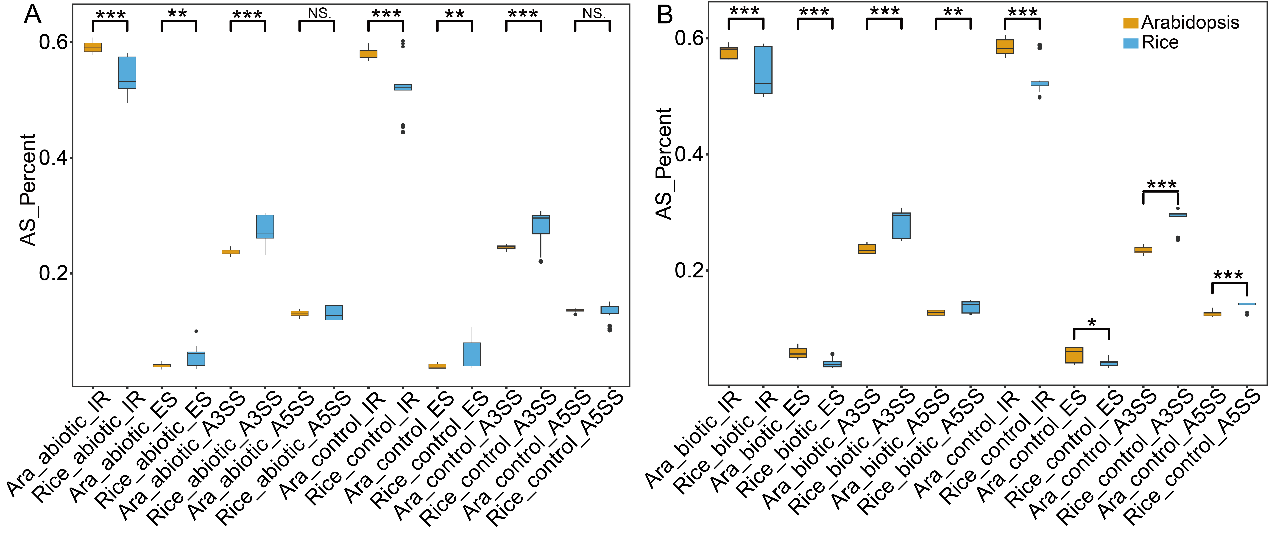


Fig S2. The percentage of IR, ES, A3SS and A5SS events in samples from Arabidopsis and rice under abiotic stress (A), biotic stress (B) and control groups.


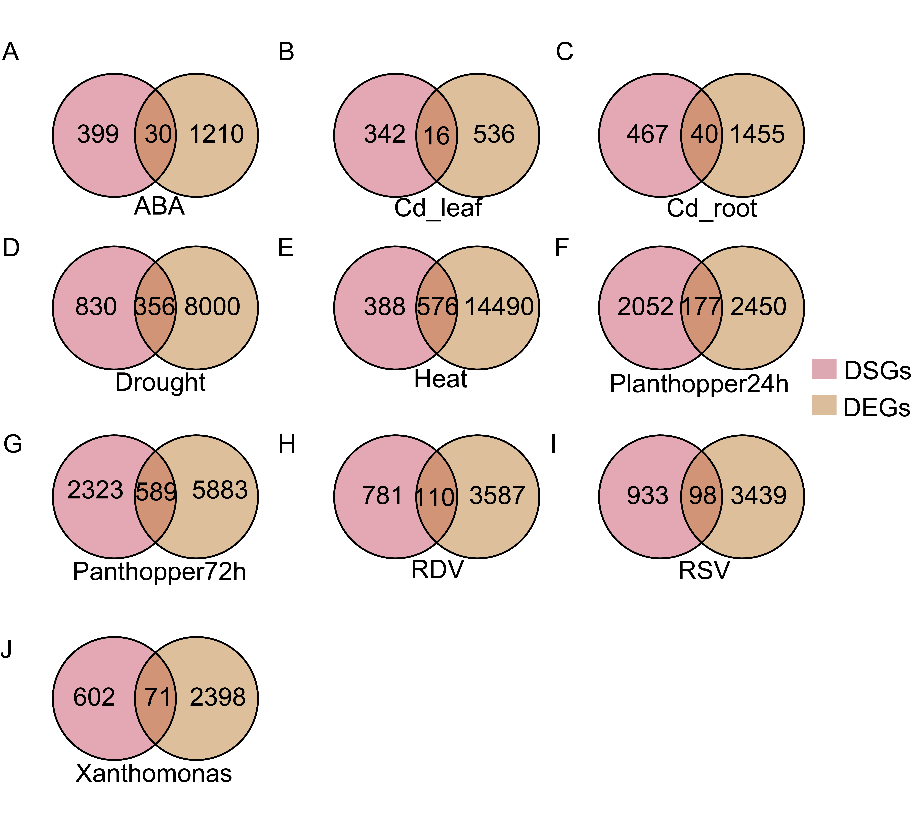


Fig S3. Venn diagram showing the overlapping DSGs and DEGs in rice under different stresses.
